# Real-world effectiveness of vortioxetine in outpatients with major depressive disorder: functioning and dose effects

**DOI:** 10.1186/s12888-022-04109-5

**Published:** 2022-08-12

**Authors:** Eugenia Papalexi, Andreas Galanopoulos, Dimitrios Kontis, Maria Markopoulou, Georgia Balta, Evaggelos Karavelas, Panagiotis Panagiotidis, Themistoklis Vlachos, Anders Ettrup

**Affiliations:** 1 Lundbeck Hellas, 109 Kifisias Avenue & Sina, 15124 Maroussi, Athens Greece; 2 4th Psychiatric Department, Psychiatric Hospital of Attica, Athens, Greece; 3 Department of Forensic Psychiatry, Psychiatric Hospital of Thessaloniki, Stavroupolis, Thessaloniki, Greece; 4 Department of Pharmacology, Medical School, National and Kapodistrian University of Athens, Athens, Greece; 5 Ensynaisthisis, Thessaloniki, Greece; 6 Department of Psychiatry, 424 General Military Hospital of Thessaloniki, Thessaloniki, Greece; 7 Department of Psychiatry, 251 Airforce Military Hospital, Athens, Greece; 8 H. Lundbeck A/S, Valby, Denmark

**Keywords:** Major depressive disorder (MDD), Functioning, Vortioxetine, Real-world effectiveness, Dose-response

## Abstract

**Background:**

Functional recovery is an important treatment goal in major depressive disorder (MDD). This study assessed the real-world effectiveness of vortioxetine in patients with MDD, with particular focus on functioning; dose–response was also assessed.

**Methods:**

This was a non-interventional, prospective, multicenter study conducted in Greece. Adult outpatients with MDD (*n* = 336) initiating vortioxetine (5–20 mg/day flexible dosing) as treatment for a current major depressive episode were followed for 3 months. Analyses were stratified according to vortioxetine dosage at 3 months: 5–10 mg/day versus 15–20 mg/day. Functioning was assessed using the Sheehan Disability Scale (SDS).

**Results:**

Mean ± standard error SDS total score decreased (improved) from 18.7 ± 0.3 at baseline to 12.9 ± 0.3 after 1 month of vortioxetine treatment and 7.8 ± 0.4 after 3 months (*p* < 0.001 vs. baseline for all comparisons). Functional recovery (SDS score ≤ 6) was achieved in 14.6% of patients after 1 month of treatment and 48.4% of patients after 3 months. Improvement from baseline in SDS total and domain scores at 3 months was more pronounced in patients receiving vortioxetine 15–20 mg/day than in those receiving vortioxetine 5–10 mg/day. The mean ± standard error change in SDS total score from baseline was 9.2 ± 0.8 in the 5–10 mg/day group and 12.1 ± 0.4 in the 15–20 mg/day group (*p* < 0.001). Limitations of this study include its non-interventional study design and lack of a control group or active comparator.

**Conclusions:**

Statistically significant and clinically relevant improvements in functioning were seen in patients with MDD treated with vortioxetine in a real-world setting. Higher doses of vortioxetine were associated with significantly greater improvements in functioning.

**Supplementary Information:**

The online version contains supplementary material available at 10.1186/s12888-022-04109-5.

## Introduction

Major depressive disorder (MDD) is a common and debilitating condition estimated to affect more than 160 million people worldwide [1]. MDD is a multidimensional disease characterized by emotional, cognitive, and somatic symptoms that significantly compromise functioning [2]. Functional recovery is the current treatment goal for patients with MDD [3–6]; however, functional impairment may persist even after resolution of other symptoms [7]. Residual functional impairment following remission of mood symptoms in patients with MDD has been shown to be a predictor of subsequent relapse [8].

Vortioxetine is a multimodal antidepressant with a unique mechanism of action [9]. It acts both as an inhibitor of the serotonin transporter and as a modulator of several serotonin receptor subtypes, thereby directly and indirectly influencing neurotransmitter systems relevant to the neurobiology of depression, including serotonin, noradrenaline, dopamine, acetylcholine, histamine, glutamate, and gamma-aminobutyric acid systems [9–11]. First approved by the US Food and Drug Administration for the treatment of adults with MDD in 2013, vortioxetine has since been licensed in more than 80 countries worldwide. Vortioxetine has an approved therapeutic dose range of 5–20 mg/day [12, 13]. In most countries, the recommended starting dose of vortioxetine in adult patients with MDD is 10 mg once daily. Depending on individual patient response, the dosage of vortioxetine may be increased to a maximum of 20 mg/day or decreased to a minimum of 5 mg/day.

Early optimized antidepressant dosing is likely to afford the best possible treatment outcomes [3, 5]. Indeed, subtherapeutic dosing has been shown to contribute to early withdrawal from antidepressant treatment in patients with MDD [14]. A recent analysis of more than 50,000 patients who were prescribed selective serotonin reuptake inhibitors (SSRIs) for the treatment of depression found that 60% never received the treatment dose reported to exert maximum antidepressant effect [15]. However, finding the right dose for each patient can be challenging in routine practice. A dose–response relationship for antidepressant efficacy remains controversial [16–19], and individual patients may respond differently to the same drug dosage. Nevertheless, it would be helpful to provide clinicians with meaningful guidance on how to optimize antidepressant dosage so that patients receive maximum benefit from their treatment.

Vortioxetine is one of the few antidepressants with a dose–response relationship demonstrated in fixed-dose, randomized controlled clinical trials [20, 21]. The present study was undertaken to assess the real-world effectiveness of vortioxetine for the treatment of patients with MDD in Greece, with particular focus on the effect of treatment on patients’ functioning. The impact of vortioxetine dosage on treatment response was also assessed.

## Methods

### Study design

This was a 3-month, non-interventional, prospective, multicenter study conducted in private psychiatric offices or in public hospitals in Greece. Participants were adult (aged ≥ 18 years) outpatients with MDD who were initiating vortioxetine as first- or second-line treatment for a current major depressive episode at their physician’s discretion. Vortioxetine was administered at a flexible dosage of 5–20 mg/day according to local prescribing information [13]. In Greece, the starting and recommended dosage of vortioxetine in adults aged < 65 years is 10 mg/day. According to the prescribing information, vortioxetine may be increased to a maximum of 20 mg or decreased to a minimum of 5 mg once daily *“depending on individual patient response”* [13]. Consequently, healthcare providers were free to adjust the vortioxetine dosage for each patient based on their own clinical practice and the patient’s individual needs. Vortioxetine treatment was not free for the study participants; patients were required to meet the usual prescription charges for their medication. Patients with schizophrenia or any other psychotic disorder, bipolar disorder, substance-use disorders, dementia or other neurodegenerative diseases significantly impacting cognitive functioning, or mood disorders due to an underlying general medical condition were excluded from study participation. Patients considered at significant risk of suicide or who had attempted suicide within the last 6 months were also excluded.

Study assessments were conducted at baseline and after 1 and 3 months of vortioxetine treatment. The study was conducted in accordance with the Declaration of Helsinki and Good Clinical Practice guidelines. All experimental protocols were approved by the institutional review boards at the two coordinating hospitals (Psychiatric Hospital of Attica and Psychiatric Hospital of Thessaloniki). At the time the study was conducted, ethical approval for observational studies was not required at a national level. All patients provided written informed consent for participation.

### Study objectives

The primary objective of this study was to assess the real-world effectiveness of vortioxetine on patient functioning (measured using the Sheehan Disability Scale [SDS]) and the rate of functional recovery for the total treatment duration, as well as the impact of baseline patient and MDD characteristics on the change in patient functioning (difference in SDS total score) after 3 months of vortioxetine treatment. Secondary objectives included assessment of the effectiveness of vortioxetine on depression severity (assessed by the Montgomery–Åsberg Depression Rating Scale [MADRS] and the 9-item Patient Health Questionnaire [PHQ-9]), cognitive symptoms (assessed using the 20-item Perceived Deficits Questionnaire–Depression [PDQ-D-20]), and patients’ general clinical condition (assessed using the Clinical Global Impression–Severity [CGI-S] scale) for the total treatment duration, i.e. at baseline and 1 and 3 months post baseline. The effect of vortioxetine dosage on patient functioning (SDS change/difference) and severity of mood symptoms (MADRS change/difference) after 3 months of treatment was also assessed. Safety assessment was not a primary objective of this study; however, adverse events leading to withdrawal from treatment were recorded.

### Patient-reported outcomes

Patients assessed their functioning using the SDS [22, 23]. This brief self-report measure assesses functional impairment over the previous 7 days across three domains: work/school, social life/leisure, and family/home life. The level of impairment for each domain is rated using a visual analog scale ranging from 0 (not at all) to 10 (very severe). Scores from the individual domains are combined to generate the SDS total score, ranging from 0 (unimpaired) to 30 (highly impaired). In this study, all patients completed all sections of the SDS. Functional recovery was defined as an SDS total score ≤ 6 [23, 24].

Patients also rated the severity of their depressive symptoms using the PHQ-9 [25] and the severity of cognitive symptoms using the PDQ-D-20 [26, 27].

### Clinician-rated outcomes

Clinicians evaluated depression severity using the MADRS [28] and the CGI-S [29, 30]. The MADRS anhedonia factor score was also calculated (i.e. the sum of MADRS items 1, 2, 6, 7, and 8) [31, 32]. MADRS total score cut-offs for mild, moderate, and severe depression were < 20, 20–34, and ≥ 35 points, respectively [33]. Response was defined as ≥ 50% improvement in MADRS total score from baseline; remission was defined as MADRS total score ≤ 12 points.

### Statistical methods

Sample size estimation was based on calculation of the estimated 95% confidence interval (CI; normal approximation) for the change in SDS score (primary study outcome) after 3 months of vortioxetine treatment. In the open-label AtWoRC study [34], the mean ± standard deviation (SD) change (improvement) in SDS total score after 3 months of vortioxetine treatment was approximately 10.0 ± 9.0 points. The accuracy of the corresponding 95% CI was assumed to be 10% of the above-mentioned SD, which is 0.9 (i.e. almost 1 point on the SDS scale), giving a required sample size of 385 patients. In the present study, the corresponding SD of mean change in SDS total score was 0.7; thus, the study provided higher precision than that required based on the sample size calculation.

All enrolled patients who met the study inclusion criteria were included in this analysis. All analyses were conducted on observed cases (i.e. patients with missing data for particular variables were excluded from the corresponding analyses). For the dose–response analysis, patients were stratified into two groups according to their vortioxetine dosage at the end of the 3-month study period (5–10 mg/day vs. 15–20 mg/day).

Categorical variables are presented as number (%), while continuous variables are reported as means and SD or standard error (SE). For all study outcomes (SDS, MADRS, PHQ-9, PDQ-D-20, and CGI-S), the effectiveness of vortioxetine over the total treatment duration was assessed using repeated measures analysis of variance with Greenhouse–Geisser correction [35]. For each of these variables, if the corresponding *p*-value was < 0.05, a paired *t* test was used to assess the pairwise differences between visits (Bonferroni correction). Cochran’s Q test was used to assess the change in the percentage of patients achieving functional recovery (i.e. SDS score ≤ 6) over time. If the corresponding *p*-value was < 0.05, McNemar’s test was used to assess the three pairwise comparisons between visits (Bonferroni correction).

The impact of baseline characteristics on the real-world effectiveness of vortioxetine on patient functioning (measured by SDS) was assessed using multiple linear regression (stepwise selection), with patient demographic characteristics, MDD history, MDD management, MDD severity (baseline MADRS total score), and baseline SDS total score as independent variables, and the difference in SDS score between the third minus the first (baseline) visit as the dependent variable. The effect of vortioxetine dosage on the change in SDS and MADRS scores after 3 months of treatment was also assessed using multiple linear regression (stepwise selection), with difference in score between the third minus the first (baseline) visit as the dependent variable. The corresponding baseline score (SDS or MADRS), vortioxetine dosage, and patient/MDD characteristics were used as explanatory variables.

All statistical analyses were performed using R version 3.6.3 [36]. For all statistical tests, the significance level was set at 0.05. All *p*-values should be considered nominal.

## Results

### Study population

Between January 15 and September 18, 2019, 337 patients were enrolled into the study, 336 of whom were included in this analysis; one patient was excluded due to missing baseline data. Baseline demographics and clinical characteristics are shown in Table 1. The mean ± SD age of study participants was 47.9 ± 14.3 years, and almost two-thirds (64.3%) were female. Over half of all patients had received higher education (53.3%) and 56.0% were in full- or part-time employment. This was the first depressive episode for approximately half of all patients (48.5%). Mean ± SD duration of MDD was 3.5 ± 6.1 years.

**Table 1:** Baseline demographics and clinical characteristics (observed cases)

Characteristic	Total (*n* = 336)
Age, years	47.9 ± 14.3
Sex, *n* (%)	
Female	216 (64.3)
Male	120 (35.7)
Educational level, *n* (%)	
Primary education	24 (7.1)
Secondary education	133 (39.6)
Higher education	179 (53.3)
Working status, *n* (%)	
Working full-time	139 (41.4)
Working part-time	49 (14.6)
Unable to work due to depression/anxiety	12 (3.6)
Unable to work due to reasons other than depression	8 (2.4)
Seeking work	17 (5.1)
Not working by choice	111 (33.0)
Living status, *n* (%)	
Living with a partner	274 (81.5)
Living alone	62 (18.5)
Newly diagnosed, *n* (%)	182 (54.2)
Family history of MDD, *n* (%)	108 (32.1)
MDD duration, years	3.5 ± 6.1
No. of depressive episodes, *n* (%)	
1	163 (48.5)
2	100 (29.8)
≥ 2	73 (21.7)

*MDD* major depressive disorder

Values are mean ± standard deviation unless otherwise indicated

Vortioxetine dosage at each visit is shown in Table 2. At baseline, most patients were receiving vortioxetine 5–10 mg/day (89.6%). Of the 311 patients who completed the 3-month visit, 200 (64.3%) were receiving vortioxetine 15–20 mg/day. Twenty-six patients (7.7%) discontinued treatment with vortioxetine (nine by the second study visit at 1 month and the remaining 17 by the final study visit at 3 months). The most common reasons for treatment discontinuation were lost to follow-up (*n* = 10), lack of efficacy (*n* = 5), and adverse events (*n* = 4; nausea/vomiting in all cases).

**Table 2:** Vortioxetine dosage at each study visit (observed cases)

Vortioxetine dosage	Patients (%)		
	**Baseline** **(*n*** **= 317)**^**a**^	**Month 1** **(*n*** **= 329)**	**Month 3** **(*n*** **= 311)**
5–10 mg/day	89.6	58.7	35.7
15–20 mg/day	10.4	41.3	64.3

^a^Vortioxetine dose data not available for all patients at baseline

### Effect on patient functioning

**Fig. 1:**
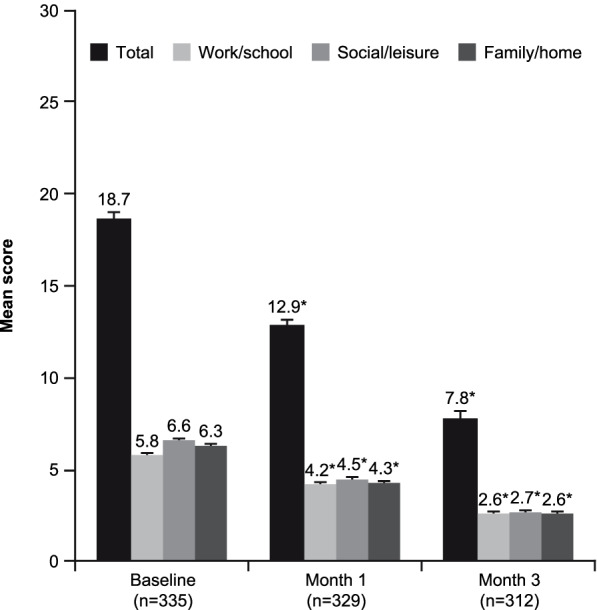
Mean (standard error) Sheehan Disability Scale total and subscale scores at each visit over the 3 months of vortioxetine treatment (observed cases). *p < 0.001 for all paired comparisons over the three visits

As shown in Fig. 1, mean ± SE SDS total score decreased (i.e. improved) significantly from 18.7 ± 0.3 at baseline to 12.9 ± 0.3 after 1 month of vortioxetine treatment and 7.8 ± 0.4 after 3 months (Greenhouse–Geisser corrected: *F* = 557, degrees of freedom [df] = 1.6, *p* < 0.001; *t* test, *p* < 0.001 for all pairwise comparisons between visits). The mean ± SE reduction in SDS total score at the third visit compared with the baseline visit was 11.0 ± 0.4 points (corresponding 95% CI: 10.3, 11.8); this represents a 58% reduction from baseline. Significant improvement was observed in all three SDS domains over the 3 months of vortioxetine treatment (work/school: *F* = 343, df = 1.6, *p* < 0.001; social life: *F* = 504, df = 1.7, *p* < 0.001; home/family life: *F* = 447, df = 1.7, *p* < 0.001; *t* test, *p* < 0.001 for all pairwise comparisons). Functional recovery (SDS score ≤ 6) was achieved in 14.6% of patients after 1 month of vortioxetine treatment and 48.4% of patients after 3 months (Cochran’s Q = 222, df = 2.0, *p* < 0.001; McNemar’s test, *p* < 0.001 for all pairwise comparisons).

SDS total score was the only baseline characteristic found to be significantly related to improvement in functioning by multiple linear regression analysis; greater improvement was seen in patients with higher baseline SDS total score (multiple linear regression coefficient B = 0.6, *t* statistic = 9.2, *p* < 0.001).

### Effect on mood and cognitive symptoms

**Fig. 2:**
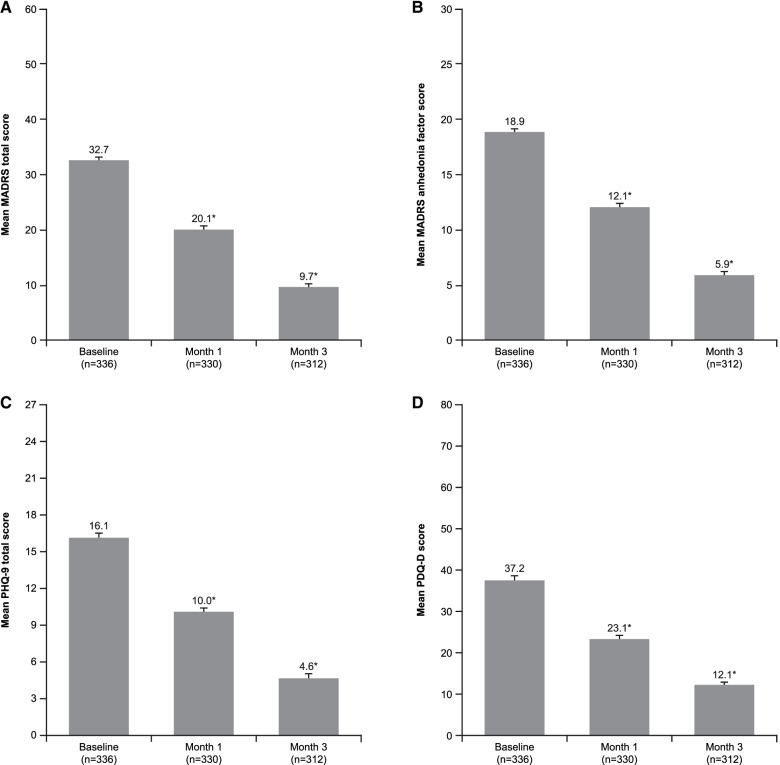
Mean (standard error) symptom assessment scale scores at each visit over the 3 months of vortioxetine treatment (observed cases): (A) MADRS total score; (B) MADRS anhedonia factor score; (C) PHQ-9 total score; and (D) PDQ-D total score. *p < 0.001 for all paired comparisons over the three visits. MADRS = Montgomery–Åsberg Depression Rating Scale; PDQ-D = 20-item Perceived Deficits Questionnaire–Depression; PHQ-9 = 9-item Patient Health Questionnaire

**Fig. 3:**
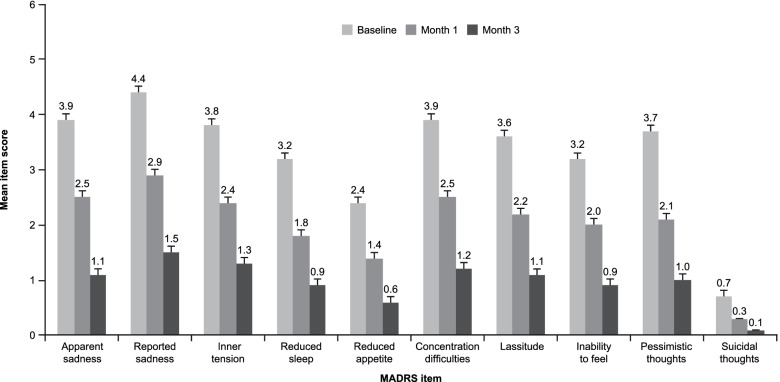
Mean (standard error) MADRS item scores over the 3 months of vortioxetine treatment (observed cases). MADRS = Montgomery–Åsberg Depression Rating Scale

Significant improvements were observed across all depression-related outcomes over the study period. Mean ± SE MADRS total score decreased from 32.7 ± 0.5 at baseline to 20.1 ± 0.6 after 1 month of vortioxetine treatment and 9.7 ± 0.5 after 3 months (Greenhouse–Geisser correction *F* = 1318, df = 1.7, *p* < 0.001; *t* test, *p* < 0.001 for all pairwise comparisons between visits); a decrease of 23.4 ± 0.5 points at the third visit compared with the baseline visit (Fig. 2A). The mean ± SE MADRS anhedonia factor score decreased from 18.9 ± 0.3 at baseline to 12.1 ± 0.3 after 1 month of vortioxetine treatment and 5.9 ± 0.3 after 3 months (*F* = 16,469, df = 1.7, *p* < 0.001; *t* test, *p* < 0.001 for all pairwise comparisons), a decrease of 13.2 ± 0.3 points at the third visit compared with the baseline visit (Fig. 2B). This corresponds to a 72% reduction from baseline in MADRS total score and a 70% reduction in MADRS anhedonia factor score over the 3 months of vortioxetine treatment. Improvements were observed across all individual MADRS items, suggesting that vortioxetine had broad efficacy across the spectrum of depressive symptoms (Fig. 3).

After 1 month of vortioxetine treatment, 34.2% of patients were classed as MADRS responders (i.e. had experienced ≥ 50% improvement in MADRS total score) and 25.2% achieved symptomatic remission (i.e. achieved MADRS total score ≤ 12 points). Corresponding rates of response and remission after 3 months of vortioxetine treatment were 84.3% and 67.6%, respectively.

The mean ± SE CGI-S score improved from 4.7 ± 0.1 (markedly ill) at baseline to 3.5 ± 0.1 (moderately–mildly ill) at 1 month and 2.2 ± 0.1 (borderline ill) at 3 months (Greenhouse–Geisser correction *F* = 922, df = 1.7, *p* < 0.001; *t* test, *p* < 0.001 for all pairwise comparisons between visits). The proportion of patients classed as not at all ill or borderline ill according to CGI-S score was 18.5% after 1 month of vortioxetine treatment and 64.6% after 3 months.

Over the 3 months of vortioxetine treatment, patients reported a 71% reduction from baseline in the severity of depressive symptoms assessed by the PHQ-9 (Fig. 2C) and a 68% reduction in perceived cognitive symptoms assessed by the PDQ-D-20 (Fig. 2D).

### Effect of vortioxetine dosage

**Fig. 4:**
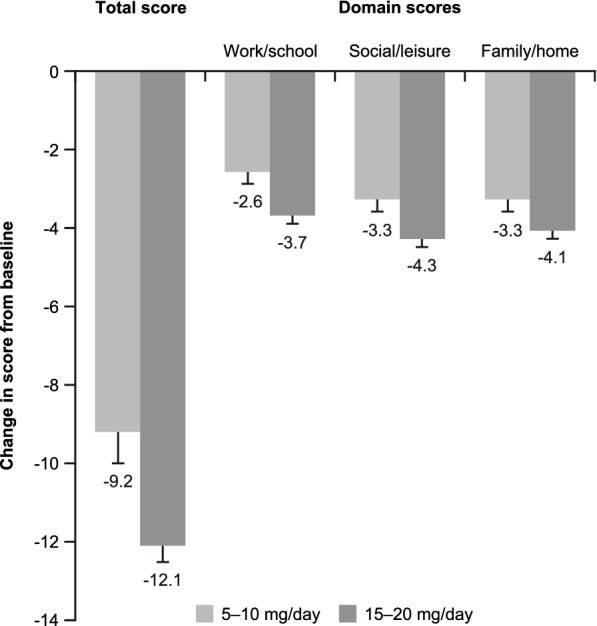
Reduction in mean (standard error) Sheehan Disability Scale total and domain scores from baseline after 3 months of vortioxetine treatment according to vortioxetine dose at study end (observed cases). *p* < 0.001 for repeated measures analysis of variance Greenhouse–Geisser F and *t* test for all paired comparisons between the three visits

Baseline patient characteristics according to vortioxetine dosage at 3 months are shown in Supplementary Table 1 (see Additional file 1). Improvement in SDS total score from baseline was more pronounced in patients receiving vortioxetine 15–20 mg/day at 3 months than in those receiving vortioxetine 5–10 mg/day, and this dose–response relationship was apparent across all SDS domains (Fig. 4). The improvement in mean ± SE SDS total score was 9.2 ± 0.8 in the 5–10 mg/day group and 12.1 ± 0.4 in the 15–20 mg/day group (multiple linear regression, B = 0.6, *t* statistic = 9.1, *p* < 0.001). Significantly greater improvements were seen in patients receiving vortioxetine 15–20 mg/day versus 5–10 mg/day for all SDS domains. The improvement in the work/school domain score was 2.6 ± 0.3 in the 5–10 mg/day group versus 3.7 ± 0.2 in the 15–20 mg/day group (B = 0.8, *t* statistic = 3.1, *p* = 0.002). Respective improvements were 3.3 ± 0.3 versus 4.3 ± 0.2 for the social life domain score (B = 0.7, *t* statistic = 2.8, *p* = 0.005), and 3.3 ± 0.3 versus 4.1 ± 0.2 for the family/home life domain score (B = 0.7, *t* statistic = 2.6, *p* = 0.009). After 3 months of vortioxetine treatment, the percentage reduction in SDS total score from baseline was 63% in patients receiving vortioxetine 15–20 mg/day compared with 51% in those who were receiving vortioxetine 5–10 mg/day. After 3 months of vortioxetine treatment, functional recovery (SDS score ≤ 6) was achieved in 50.0% of patients receiving vortioxetine 15–20 mg/day and 45.9% of those receiving vortioxetine 5–10 mg/day.

The improvement in mean ± SE MADRS total score from baseline over the 3 months of vortioxetine treatment was numerically greater in patients receiving vortioxetine 15–20 mg/day than in those receiving vortioxetine 5–10 mg/day (24.5 ± 0.7 vs. 21.5 ± 0.8 points, respectively); however, this difference was not found to be significant using multiple linear regression stepwise analysis. The only parameter that entered the model was MADRS baseline score: patients with more severe MDD at baseline showed greater MADRS improvement. However, physicians prescribed the higher dosage to patients with higher MADRS scores at baseline (mean ± SE MADRS total score at baseline, 34.8 ± 0.8 in patients receiving 15–20 mg/day vs. 29.8 ± 0.9 in those receiving 5–10 mg; *t* test statistic=-4.6, df = 309, *p*-value < 0.001).

## Discussion

Broad improvements in symptoms of depression were seen in patients with MDD treated with vortioxetine in real-world settings in this study, with statistically significant and clinically relevant improvements observed in patient-reported functioning assessed using the SDS. Patients reported similar improvements across all three domains of the SDS (work/school, social life/leisure, and family/home life). Functional recovery (defined as an SDS score ≤ 6) was achieved in approximately 15% of patients after 1 month of vortioxetine treatment and approximately 50% of patients after 3 months. Functional recovery is increasingly recognized to be an important treatment goal in patients with MDD [3–6]. Symptomatic response (defined as ≥ 50% improvement in MADRS total score from baseline) was achieved by almost 85% of patients after 3 months of vortioxetine treatment, with just over two-thirds of all patients achieving remission from depressive symptoms by the end of follow-up (i.e., MADRS total score ≤ 12 points). Our findings are in line with results of a meta-analysis of data from nine short-term, randomized controlled studies showing improved overall functioning and high rates of functional remission in adults with MDD treated with vortioxetine 5–20 mg/day [37].

Broad therapeutic efficacy of vortioxetine in patients with MDD has been demonstrated in other recently completed real-world studies [34, 38–40]. In another recent 24-week, observational, prospective cohort study of similar design in outpatients with MDD initiating treatment with vortioxetine in routine care settings in Canada, France, Italy and the USA (the RELIEVE study), clinically relevant improvements in overall functioning, depressive symptoms, cognitive symptoms and performance, and health-related quality of life were reported over the 6-month treatment period [40]. Of note, greater improvement in functioning as assessed by mean change in SDS total score was seen after 3 months of vortioxetine treatment in the present study than in the multinational RELIEVE study (-11 versus -7 points, respectively) [40]. This may be due to differences in patient population, treatment line, or vortioxetine dosage. For example, in the present study, almost half of all patients were experiencing their first depressive episode compared with only 20% of those in the RELIEVE study. In RELIEVE, the greatest clinical benefits were achieved when vortioxetine was used as a first-line treatment [40].

The observed improvements in self-reported cognitive symptoms during treatment with vortioxetine in the present study are consistent with results of previous studies [34, 38, 41–46]. It seems reasonable to assume that the positive impact of vortioxetine on functioning in patients with MDD is at least in part due to the beneficial effect of treatment on cognitive symptoms. In a study in patients with MDD who were either initiating or undergoing their first switch of antidepressant monotherapy, patient-reported cognitive symptoms (assessed using the shorter 5-item Perceived Deficits Questionnaire) were found to be independently associated with patient functioning throughout the 2 years of follow-up [47, 48].

In the present study, few patients (1.2%) discontinued treatment due to adverse events (nausea/vomiting in all cases), and no new safety concerns were identified. A pooled analysis of safety and tolerability data from 11 randomized, double-blind, placebo-controlled, fixed-dose, short-term studies also found nausea and vomiting to be the most common dose-related treatment-emergent adverse events associated with vortioxetine, with incidence plateauing at a dosage of 15 mg/day [49].

A recent systematic literature review and network meta-analysis found no evidence of a dose–response relationship for antidepressant efficacy of SSRIs [19]. In contrast, available clinical trial data show vortioxetine to have dose-dependent efficacy across the therapeutic dose range [20, 21, 50]. In particular, results of a pooled analysis of 11 short-term, double-blind, randomized, placebo-controlled studies of vortioxetine in patients with MDD, which included MADRS and SDS assessments, demonstrated significant dose-dependent improvements in functioning and overall depressive symptoms (including anhedonia) in vortioxetine-treated patients compared with those who received placebo [32].

In the present study, both overall improvement in functioning and the proportion of patients who achieved functional recovery were found to be higher in the cohort receiving vortioxetine 15–20 mg/day than in that receiving lower dosages (5–10 mg/day). The observed dose–response effect for improvement in depressive symptom severity did not attain statistical significance; however, this was most likely due to the fact that clinicians tended to use higher doses of vortioxetine from the start of treatment in patients with more severe symptoms at baseline. In all, almost two-thirds of patients were receiving the maximum therapeutic dosage of vortioxetine (15–20 mg/day) at the end of the 3-month treatment period, which is consistent with the results of other flexible-dose clinical trials of vortioxetine in patients with MDD [39, 42, 46, 51]. Vortioxetine was not widely used in routine clinical practice at the time these studies were undertaken; with increased experience and evidence, clinicians may be more likely to increase patients’ vortioxetine dosage for early optimization of clinical response.

Interpretation of these findings should take into account the inherent limitations of the naturalistic and observational study design, and the lack of control group or active comparator. In addition, we cannot rule out potential for bias caused by clinicians overestimating the effects of vortioxetine on patients’ depressive symptoms when administering the MADRS and CGI-S. However, it should be noted that the positive effects of vortioxetine on depressive symptoms were confirmed by patients using self-reported questionnaires (PHQ-9 and PDQ-D-20), supported by the observed improvement in patient functioning (assessed using the SDS). As such, the consistent effects of vortioxetine across the spectrum of depressive symptoms and functioning measures were unlikely to have occurred by chance. A further possible limitation is that patients with MDD generally require long-term treatment and patients in this study were followed for only 3 months. However, in another observational study in outpatients with MDD receiving treatment with vortioxetine in routine care settings in Canada, France, Italy and the USA, the significant improvements observed in overall functioning, depressive symptoms, cognitive symptoms and performance, and health-related quality of life that were achieved within 12 weeks of treatment initiation (i.e., at the first post-baseline assessment time point) were sustained over a period of 6 months [40]. In addition, we did not assess cognition using objective neurocognitive tests. However, the improvement in cognitive symptoms reported by patients using the PDQ-D-20 was indirectly confirmed by the observed improvement in the clinician-administered MADRS item that measures concentration difficulties. Finally, the analysis of the effects of vortioxetine dosage was based on pooled dosages (5–10 and 15–20 mg/day) rather than individual doses (5, 10, 15, or 20 mg/day). Important strengths of this study include the real-world setting, the broad inclusion criteria, the relatively large patient population, and the use of both patient-reported and clinician-rated outcome measures to assess symptom severity and functioning.

In summary, in this study, statistically significant and clinically relevant improvements in patient-reported functioning were seen in patients with MDD treated with vortioxetine in a real-world setting. Higher dosages of vortioxetine were associated with significantly greater improvements in functioning and higher rates of functional recovery. Our findings suggest that vortioxetine dosage can be increased to 20 mg/day early in the course of treatment in patients with MDD in order to achieve optimal therapeutic benefit and promote functional recovery. The observed dose–response relationship allows prescribers to increase vortioxetine dosage to optimize clinical response in patients with MDD as a possible alternative to switching to another antidepressant medication.

## Supplementary Information

**Additional file 1.**

## Data Availability

The authors confirm that the data supporting the findings of this study are included in this article and its additional supplementary information files. The authors may be contacted for further data sharing.

## Abbreviations

CGI-S: Clinical Global Impression–Severity
CI: confidence interval
df: degrees of freedom
MADRS: Montgomery–Åsberg Depression Rating Scale
MDD: major depressive disorder
PDQ-D-20: 20-item Perceived Deficits Questionnaire–Depression
PHQ-9: 9-item Patient Health Questionnaire
SD: standard deviation
SDS: Sheehan Disability Scale
SE: standard error
SSRI: selective serotonin reuptake inhibitor
