# Supplementary material for: Real-world effectiveness of vortioxetine in outpatients with major depressive disorder: functioning and dose effects
Source: BMC Psychiatry. 2022 Aug 12;22:548. doi: 10.1186/s12888-022-04109-5 (PMC9373318; doi:10.1186/s12888-022-04109-5)
Supplement: Supplementary file 1 — Additional file 1. [file 12888_2022_4109_MOESM1_ESM.pdf]

Supplementary information

Supplementary Table 1 Baseline patient demographics and clinical characteristics according to vortioxetine dosage at 3 months (observed cases)

Characteristic	Vortioxetine daily dosage at 3 months		
	**5–10 mg**	**15–20 mg**	**Total**
No. of patients	111	200	311
Age, years	46.7±15.6	48.4±13.5	47.8±14.3
Sex, *n* (%)			
Female	71 (64.0)	128 (64.0)	199 (64.0)
Male	40 (36.0)	72 (36.0)	112 (36.0)
MDD duration, years	2.7±5.2	3.7±6.3	3.4±6.0
SDS total score at baseline	18.1±5.5	19.2±5.0	18.8±5.2
MADRS total score at baseline	29.8±9.3	34.8±9.0	33.0±9.4
MDD severity at baseline, *n* (%)			
Mild (MADRS total score <20)	15 (13.5)	6 (3.0)	21 (6.8)
Moderate (MADRS total score 20–34)	62 (55.9)	93 (46.5)	155 (49.8)
Severe (MADRS total score ≥35)	34 (30.6)	101 (50.5)	135 (43.4)

MADRS = Montgomery–Åsberg Depression Rating Scale; MDD = major depressive disorder; SDS = Sheehan Disability Scale.

Values are mean±standard deviation unless otherwise indicated.
